# Locally invasive cholangiocarcinoma causing gastric outlet obstruction in heterotaxy syndrome: A case report and review of literature

**DOI:** 10.1016/j.radcr.2023.10.050

**Published:** 2023-11-22

**Authors:** Wanyang Qian, Benjamin M. Mac Curtain, James P. Ryan, Suresh Navadgi

**Affiliations:** a Department of Surgery, St John of God Subiaco Hospital, Subiaco, Western Australia; b Department of Radiology, St Vincent's University Hospital, Dublin, Ireland

**Keywords:** Partial heterotaxy, Gastrojejunostomy, Anatomical variant, Gastric outlet obstruction

## Abstract

Heterotaxy syndrome is a disease of embryo development resulting in abnormal distribution of thoracic and abdominal organs across the left-right axis. In this case, A 77-year-old gentleman was admitted with gastric outlet obstruction secondary to cholangiocarcinoma. This is on a background of heterotaxy syndrome, specifically *situs ambiguus*. The patient's anatomical variations included a right-sided stomach, midline liver, and asplenia. Due to variant anatomy and risk of aspiration; endoscopy was abandoned in favor of surgical bypass via a gastrojejunostomy. Although technically challenging, complex upper abdominal surgery in heterotaxy syndrome has been described in the literature. Variations in anatomy observed in heterotaxy syndrome may render patients ineligible for resection of cholangiocarcinoma and increase the risk of complications. Careful preoperative planning with imaging and meticulous intraoperative dissection is required.

## Introduction

Heterotaxy Syndrome is a disease of embryo development resulting in abnormal lateralization and distribution of thoracic and abdominal organs. Where the normal asymmetrical arrangement of anatomy is coined *situs solitus*, heterotaxy can result in *situs inversus*; which is a complete mirror image of normal anatomy, or *situs ambiguus*; which is an incomplete mirror image. This can be further subdivided into left *(polysplenia syndrome)* and right *(asplenia syndrome)* isomerism*,* each associated with anatomical anomalies [1].

Right isomerism is the focus of this report. This process is not fully understood at an embryological level, however, disruption of the midline barrier preventing right and left lateralization involving the noctochord is thought to have a role [2]. The exact cause of heterotaxy syndrome is unclear but may be related to gene mutations such as FOXA2 or MMP21 [1,3]. There is a male preponderance, and it has been reported to occur most commonly in conjunction with a bridging liver, absent spleen, and left-sided vena cava; in a published case series [2]. Commonly, patients are also observed to have 3 lung lobes, bilaterally [4]. The absence of the coronary sinus septum is also a common finding in up to 85% of patients. As well as this, various cardiac defects are also reported [2]. Further illustrating the degree of cardiac involvement, congenital heart disease has been reported to occur in between 50% to 100% of patients. Further studies are in agreement with this reporting a cardiac defect rate of over 90% in asplenic heterotaxy [5]. Immune dysfunction may also be present due to patient asplenia [4]. Of note, this is also a cause of Howell-Jolly bodies on peripheral blood smear [6]. The incidence of *situs ambiguus* is rarer in comparison to situs inversus [7].

## Case report

A 77-year-old gentleman was admitted to our institution with a 2-month history of weight loss and fatigue on a background of unresectable cholangiocarcinoma of the distal common bile duct. He also reported a large volume of vomitus once per week consisting of stomach contents. No infective symptoms were present.

The patient's medical history included *situs ambiguus*, consisting of an atrial septal defect, right-sided stomach, thoracic esophagus, midline liver and gallbladder, truncated pancreas, left-facing duodenum from the first to the second part (D1–D2), and asplenia. He also suffered from pulmonary arterial hypertension and bilateral lower lobe pulmonary embolisms which were actively managed on therapeutic doses of low molecular weight heparin.

Significant surgical history included repair of an atrial septal defect, cardiac pacemaker insertion, cholecystectomy, right hemicolectomy for mucinous adenocarcinoma (in remission), right orchidectomy on a background of testicular cancer, pancreatectomy on a background of pancreatic adenocarcinoma and a right retroperitoneoscopic nephrectomy for atrophic kidney disease secondary to severe hydronephrosis stemming from pelvic ureteric junction obstruction.

A diagnosis of cholangiocarcinoma was made 2 years before this presentation. He was initiated on a chemotherapy regime consisting of Carboplatin and 5-Flurouracil in combination with radiotherapy. Prophylactic internalized biliary stents were placed, and duodenal stenting was performed for gastric outlet obstruction in another institution.

On admission the examination revealed stable observations and a distended abdomen with tenderness in the epigastrium. Phlebotomy demonstrated anemia (hemoglobin 85 g/L) and evidence of malnutrition with an albumin level of 17 g/L.

**Fig. 1:**
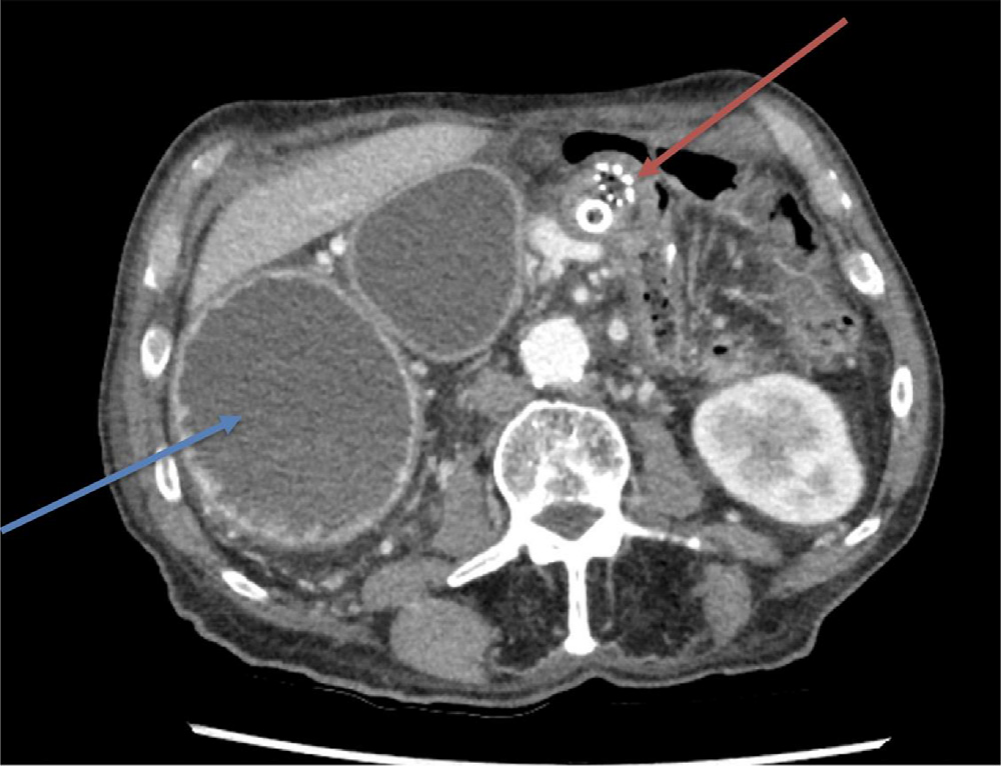
Axial slice of CT abdomen and pelvis with contrast demonstrating distended fluid-filled right-sided stomach. Red arrow - Compression of duodenal stent by soft tissue mass, likely relating to patient's history of cholangiocarcinoma. Blue arrow – Distended right-sided stomach.

**Fig. 2:**
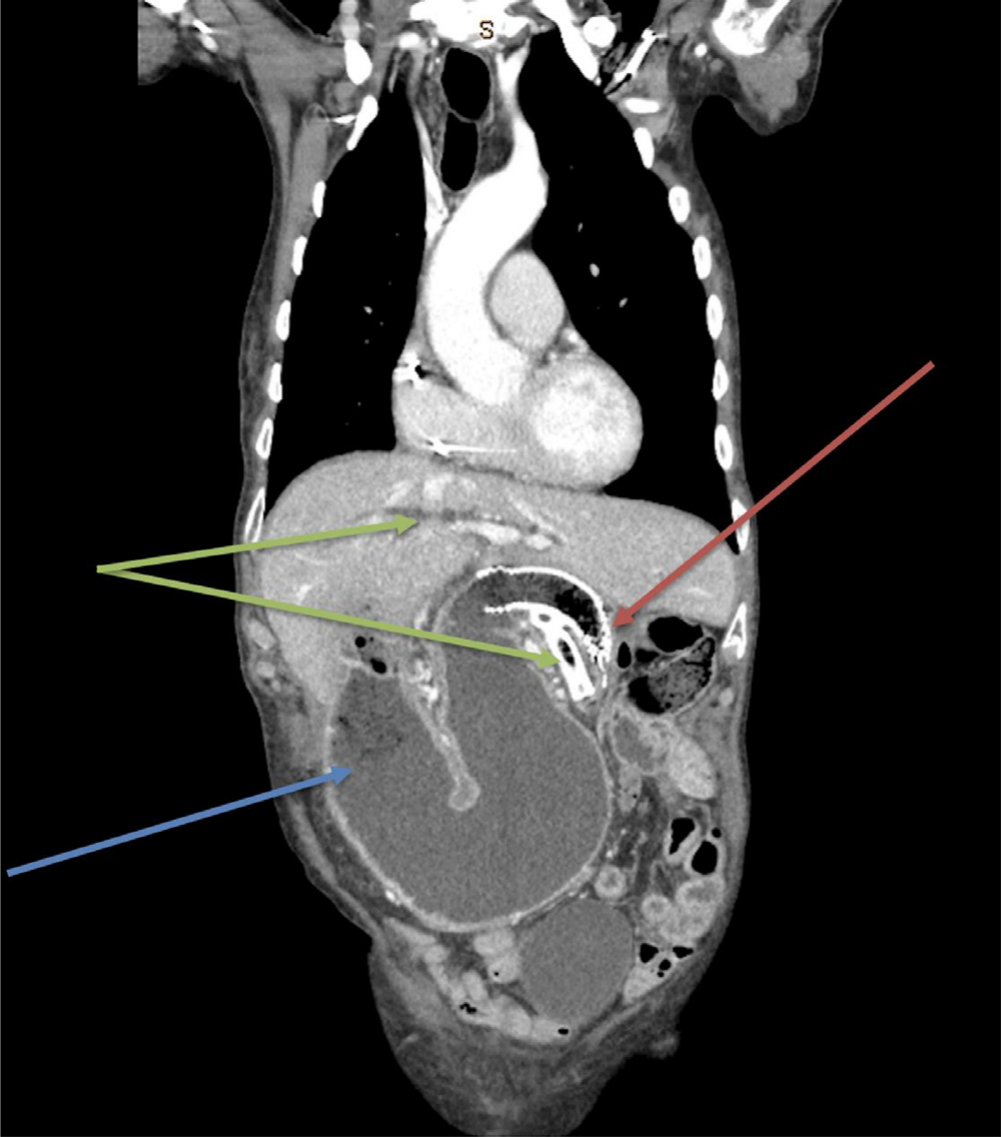
Coronal slice of CT chest, abdomen, and pelvis with contrast demonstrating distended right-sided stomach and midline liver, with duodenal and biliary stenting in-situ. Red arrow – Compression of the distal aspect of the duodenal stent. Blue arrow – Distension of the right-sided stomach to the level of the duodenal stent. Green arrows – Biliary stent in situ with intrahepatic biliary dilatation and lack of pneumobillia.

A CT abdomen pelvis with contrast was performed. The axial slices of the CT demonstrated a distended right-sided stomach secondary to compression of the duodenal stent due to a soft tissue mass related to patient's known history of cholangiocarcinoma (Fig. 1). In the coronal slices, the biliary stent is seen with new intrahepatic duct dilatation and a new absence of pneumobilia, suggesting stent occlusion (Fig. 2).

He was managed with intravenous hydration and initiated on total parenteral nutrition. An initial assessment for gastroscopy was attempted but abandoned due to aberrant anatomy and high aspiration risk. Surgical gastrojejunostomy and bypass of obstruction were made after discussion at a multidisciplinary level.

A laparotomy was performed with significant division of adhesions required. Variant anatomy was noted with a right-handed stomach and proximity of the hepatic vessels to the site of dissection. Small bowel loops were traced proximally to the duodenojejunal junction, and distally to the ileocecal valve. A gastrojejunostomy was then performed with an endoscopic stapler, completed with the placement of a Freka tube.

**Fig. 3:**
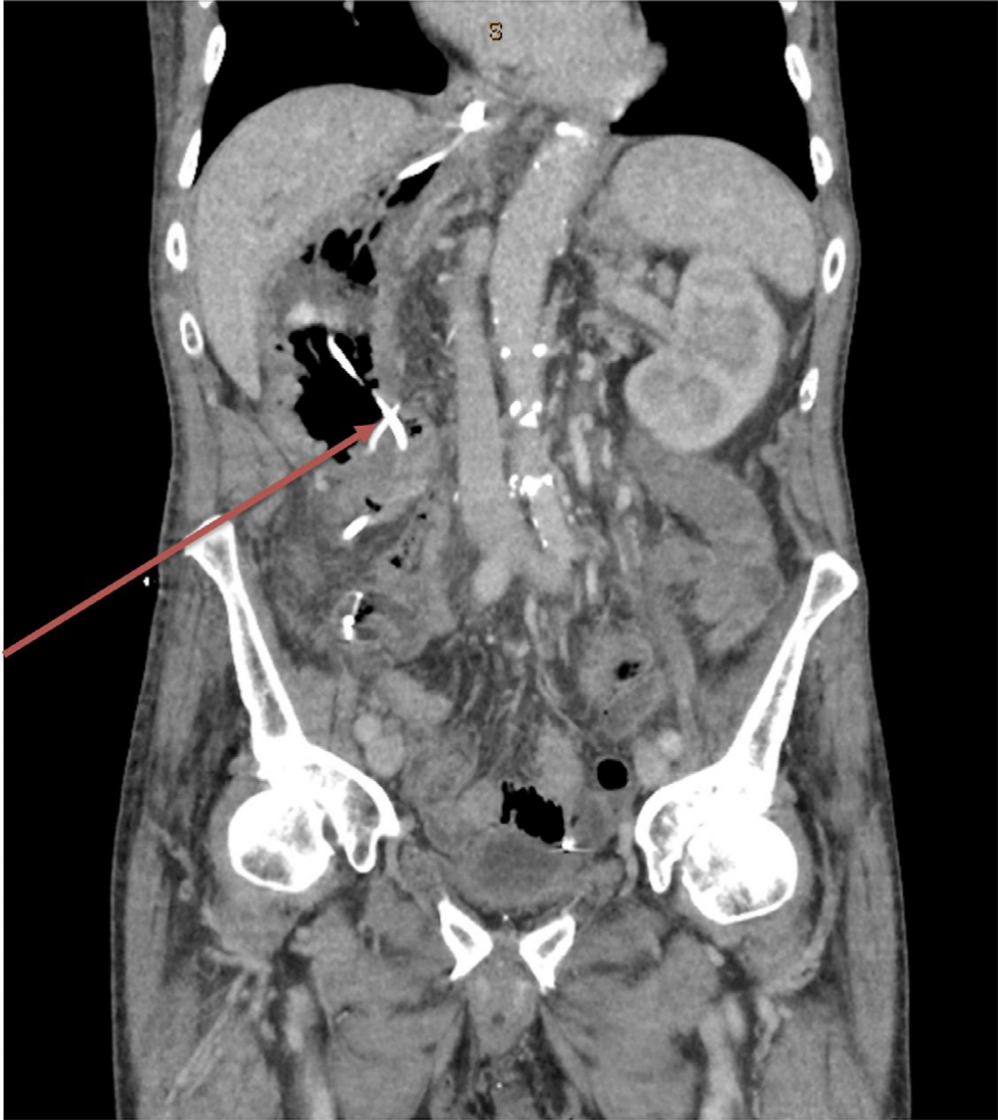
Coronal slice of CT chest, abdomen and pelvis with contrast post gastrojejunostomy, demonstrating a decompressed right-sided stomach. Red arrow – Decompressed right-sided stomach with nasojejunal tube bypassing the anastomosis.

Postoperatively, the patient was managed in intensive care for ionotropic support and nasojejunal feeding. On postoperative day 4, a CT abdomen pelvis with contrast was performed demonstrating a decompressed right-sided stomach, with a nasojejunal tube traversing the gastrojejunostomy anastomosis, without evidence of a leak (Fig. 3). The patient was gradually recommenced on oral fluids whilst total parenteral nutrition was weaned. The patient was discharged and receives ongoing surgical and oncological follow-up.

## Discussion

In heterotaxy syndrome, reporting of the anatomical variation and management options have been largely centered around the cardiovascular system, with left and right isomerism resulting in conduction abnormalities, atrial septal defect or common atrium, and variations of the systemic and pulmonary venous returns [8,9]. Cardiac management includes ventricular repair, cardiac transplantation, and ventricular palliation surgeries [8,10].

Management of abdominal manifestations of *situs ambiguus*, specifically features of right isomerism, include early childhood immunization for asplenia coupled with prophylactic antibiotics. Surgical management of obstruction secondary to intestinal malrotation, and hepatoportoenterostomy for biliary atresia is also considered [11]. Due to anatomical variations abdominal surgery is often complex in *situs ambiguus*. Upper abdominal procedures such as pancreaticoduodenectomy, hepatectomy, and liver transplantation have been described successfully in the literature [[12], [13]–14]. These studies demonstrate that surgery is feasible in patients presenting with variable anatomy with sufficient preoperative planning and meticulous dissection.

The resectability of cholangiocarcinoma is dependent on the presence of metastasis and involvement of hepatic ducts, portal vein, and hepatic arteries. Extension of tumor into hepatic vascular structures excludes patients from surgical consideration [15]. Abdominal vascular anomalies present in heterotaxy syndrome can be perilous during the resection of pancreatic and biliary tumors [16].

Cholangiocarcinoma in heterotaxy syndrome has been reported in the literature. Chirica et al. [12] detailed successful resection of cholangiocarcinoma with hilar extension. Wang et al. [17] described intrahepatic cholangiocarcinoma after undergoing a Fontan procedure in heterotaxy syndrome. To the best of the author's knowledge, no literature is present in relation to the anatomical considerations of cholangiocarcinoma in heterotaxy syndrome regarding outcomes in gastric outlet obstruction. Given the significant variation in anatomy in *situs ambiguus* it is plausible to speculate the risk of gastric outlet obstruction may be increased in heterotaxy syndrome and be attributed to a greater difficulty of surgery.

In this patient, the rare and significant anatomical anomaly along with complex surgical history mandates a multidisciplinary approach to management. The difficulty of procedures that are routinely used for investigation and management such as endoscopy are greatly increased and carry significantly increased risk in comparison to normal anatomy.

Here, the role of a radiologist is especially important, both to help plan for definitive surgical management, but also to highlight pertinent findings to the treating team. The availability of previous imaging is of paramount importance to compare and delineate new anatomy, and in our case, the absence of pneumobilia with intrahepatic duct dilation is found to be a new finding after comparison with previous imaging. This suggests occlusion of the biliary stent, which in conjunction with gastric outlet obstruction fits with the clinical picture of cholangiocarcinoma progression causing compression secondary to mass effect. Similarly, postoperative imaging is not only important to monitor progress but also acts as a new radiological baseline for comparison in the future, given the patient's complex anatomy and surgical history.

In conclusion, complex upper abdominal surgery can be performed in patients with variant anatomy such as heterotaxy syndrome. We present a case of gastric outlet obstruction secondary to cholangiocarcinoma in *situs ambiguus*. Variant anatomy in heterotaxy syndrome may exclude the feasibility of resection, add surgical complexity, and predispose patients to complications, such as gastric outlet obstruction as seen within our case presentation. Pre and postoperative imaging are especially important both for surgical planning and also as baseline imaging for the future. Previous imaging must be taken into consideration to delineate new anatomical changes. Care should be taken intraoperatively due to aberrant biliary, hepatic, and vascular anatomy, especially in the upper abdominal region.

## Patient consent

The authors declare that patient informed consent has been obtained in writing for the case report: “*Locally Invasive Cholangiocarcinoma Causing Gastric Outlet Obstruction in Heterotaxy Syndrome: A Case Report and Review of Literature*” and is reproducible on reasonable request.
